# Patient satisfaction with emergency department services in selected Saudi hospitals: a cross-sectional study

**DOI:** 10.3389/fmed.2026.1900551

**Published:** 2026-08-19

**Authors:** Maha Altewerki, Salah Alshagrawi

**Affiliations:** Department of Public Health, Saudi Electronic University, Riyadh, Saudi Arabia

**Keywords:** emergency department, healthcare quality, patient experience, patient satisfaction, patient-centered care, waiting time

## Abstract

**Objectives:**

Patient satisfaction is an important indicator of healthcare quality and patient-centered care, particularly in emergency department (ED) settings. This study aimed to assess patient satisfaction with ED services and identify service quality factors associated with overall patient satisfaction among patients attending selected government and private hospitals in Saudi Arabia.

**Methods:**

A cross-sectional study using a convenience sampling approach was conducted between April 5 and April 23, 2026, among adult patients attending the EDs of eight government and private hospitals, including five government hospitals and three private hospitals, across the Central, Western, and Eastern regions of Saudi Arabia. Data were collected using a structured questionnaire assessing satisfaction across multiple service quality domains, including waiting time, staff availability, patient involvement and communication, clarity of explanations, cleanliness, respect, and comfort. Descriptive statistics, Pearson correlation analysis, one-way analysis of variance (ANOVA), and hierarchical multiple regression analysis were performed.

**Results:**

A total of 313 participants completed the survey (response rate: 78.3%). Waiting time received the lowest satisfaction rating (*M* = 2.46, SD = 1.34), whereas staff respect received the highest rating (*M* = 3.03, SD = 1.26). Significant positive correlations were observed between all service-quality dimensions and overall satisfaction (*r* = 0.67–0.76, *p* < 0.001). Overall satisfaction differed significantly across patient-reported waiting-time categories, *F*_(2, 310)_ = 47.83, *p* < 0.001, η^2^ = 0.236, with shorter waiting times associated with higher satisfaction. In the final regression model, staff availability (β = 0.28, *p* < 0.001), comfort (β = 0.25, *p* < 0.001), and cleanliness (β = 0.19, *p* < 0.001) were significantly associated with overall satisfaction. The model explained 58% of the variance in satisfaction scores (*R*^2^ = 0.58, *p* < 0.001).

**Conclusion:**

Patient satisfaction with ED services was associated with both operational and interpersonal aspects of care. Although waiting time was the lowest-rated domain, staff availability, comfort, and cleanliness demonstrated the strongest associations with overall satisfaction. Staff availability and responsiveness, comfort, and cleanliness emerged as important domains associated with overall patient satisfaction and may represent priority targets for future quality-improvement initiatives in Saudi Arabia.

## 1 Introduction

Patient satisfaction is widely recognized as a key indicator of healthcare quality and an essential component of patient-centered care (1). It reflects patients' perceptions of the care they receive and provides valuable information regarding healthcare performance, service delivery, and organizational effectiveness. Higher levels of patient satisfaction have been associated with improved treatment adherence, greater patient trust, enhanced healthcare utilization, and better health outcomes (2–4). Consequently, healthcare systems increasingly incorporate patient satisfaction measures into quality-improvement initiatives and performance evaluation frameworks.

Although closely related, patient satisfaction, patient experience, perceived service quality, and clinical quality represent distinct constructs within healthcare research. Patient satisfaction reflects patients' subjective assessment of whether healthcare services met or exceeded their expectations (5). In contrast, patient experience refers to patients' reports of specific aspects of care, such as communication, waiting time, and interactions with healthcare professionals (6). Perceived service quality encompasses evaluations of service characteristics, including responsiveness, empathy, and the physical environment, whereas clinical quality relates to the technical appropriateness, safety, and effectiveness of medical care (5, 6).

Emergency departments (EDs) represent one of the most important points of access to healthcare services and play a critical role in the delivery of acute and urgent care. Unlike scheduled healthcare encounters, ED visits often occur under stressful circumstances characterized by pain, anxiety, uncertainty, and heightened emotional distress (7). These unique conditions make patient satisfaction particularly sensitive to both clinical and non-clinical aspects of care. Previous research has demonstrated that patient perceptions of waiting time, communication quality, staff responsiveness, empathy, and the physical care environment significantly influence satisfaction with ED services (8).

Among the factors affecting patient satisfaction in emergency settings, waiting time has consistently emerged as one of the strongest determinants. Prolonged waiting periods may increase frustration, reduce confidence in healthcare providers, and negatively affect overall perceptions of care, even when clinical outcomes are favorable (7). However, studies have shown that effective communication, respectful treatment, and clear explanations from healthcare professionals can mitigate dissatisfaction associated with waiting and contribute positively to the overall patient experience (2, 8). These findings highlight the importance of examining operational, interpersonal, and environmental dimensions of healthcare quality when evaluating patient satisfaction in EDs.

In Saudi Arabia, improving patient experience and satisfaction has become a strategic priority within the framework of Vision 2030 and the Health Sector Transformation Program. The Ministry of Health (MOH) has implemented several initiatives to promote patient-centered care and enhance healthcare quality through the systematic measurement of patient experiences and service outcomes (9). Despite these efforts, previous studies conducted in Saudi EDs have reported varying levels of patient satisfaction and have identified challenges related to waiting times, communication, and service delivery processes (10, 11). Nevertheless, most studies have focused on isolated determinants of satisfaction or have been conducted within single institutions or specific geographic regions, limiting the generalizability of their findings across different healthcare settings (13, 14, 19, 20).

Patient satisfaction in emergency departments is shaped by a complex interaction of operational, interpersonal, and environmental factors. Prolonged waiting times may influence patients' perceptions of staff responsiveness, while the quality of communication and the physical care environment may either exacerbate or mitigate dissatisfaction associated with service delays (12). Consequently, understanding how these dimensions collectively influence patient satisfaction is essential for identifying priority areas for quality improvement and developing patient-centered interventions in emergency care settings. Despite the growing body of research on emergency department satisfaction in Saudi Arabia, relatively few studies have simultaneously examined operational, interpersonal, and environmental determinants of satisfaction across different hospital sectors and geographic regions using an explicit conceptual framework (13, 14).

The present study was guided by an integrated conceptual framework combining Donabedian's structure–process–outcome model, selected dimensions of the SERVQUAL framework, and concepts from the psychology of waiting. Donabedian's model proposes that healthcare quality is influenced by structural factors, such as the physical care environment, and process-related factors, including interactions between patients and healthcare professionals (15). In emergency departments, structural characteristics include cleanliness and comfort, whereas process-related dimensions encompass waiting time, staff availability and responsiveness, patient involvement and communication, and the clarity of explanations provided to patients (15, 16). The SERVQUAL framework further highlights the importance of responsiveness, empathy, and service quality in shaping patient satisfaction (16). In addition, the psychology of waiting suggests that patients' experiences of delays are influenced not only by the duration of waiting but also by environmental conditions and the quality of communication during care (17, 18). Accordingly, the present study conceptualized cleanliness and comfort as structural factors, staff interactions and waiting experiences as process factors, and overall patient satisfaction as the outcome dimension of healthcare quality.

Previous studies conducted in Saudi Arabia have examined patient satisfaction in emergency departments using a variety of measurement approaches and study settings. Most investigations have relied on locally adapted questionnaires or standardized patient-experience instruments to evaluate dimensions such as waiting time, communication, staff behavior, and environmental conditions (14, 19). Although these studies have provided valuable insights into patient perceptions of emergency care, relatively few have simultaneously examined operational, interpersonal, and environmental determinants of satisfaction using multivariable analytical approaches. Consequently, the extent to which these dimensions collectively shape patient satisfaction within the Saudi healthcare system remains incompletely understood.

Although patient satisfaction has been extensively studied internationally, there remains a need for additional evidence from Saudi Arabia to better understand the factors that influence patients' perceptions of ED services (13, 20). Identifying patient-reported service-quality factors associated with satisfaction can help healthcare organizations develop targeted interventions to improve patient experiences and support ongoing healthcare transformation.

Therefore, this study aimed to assess patient satisfaction with emergency department services and examine how operational, interpersonal, and environmental service-quality factors, conceptualized within Donabedian's structure–process–outcome framework, are associated with overall satisfaction among patients attending selected government and private hospitals in Saudi Arabia.

## 2 Materials and methods

### 2.1 Study design

A quantitative cross-sectional study design was employed to assess patient satisfaction with ED services and identify factors associated with overall satisfaction among adult patients attending EDs in Saudi Arabia. Data were collected at a single point in time using a structured questionnaire administered following completion of the ED visit. The study was reported in accordance with the Strengthening the Reporting of Observational Studies in Epidemiology (STROBE) guidelines for cross-sectional studies.

### 2.2 Setting

The study was conducted between April 5 and April 23, 2026, in eight hospitals located in the Central, Western, and Eastern regions of Saudi Arabia. Hospitals were selected using a non-probability approach based on institutional accessibility, administrative approval, and willingness to participate. Because hospital participation depended on administrative approval and logistical feasibility, the participating institutions may differ systematically from hospitals that did not participate. Information regarding the total number of hospitals approached and the characteristics of non-participating hospitals was not systematically collected.

The participating institutions represented a mixture of public and private healthcare sectors and included facilities with varying organizational structures and patient populations. To preserve institutional confidentiality, the identities and specific characteristics of individual hospitals are not reported. Hospitals were selected to provide variation in geographic location, patient demographics, healthcare service capacity, and healthcare sector. To improve transparency, anonymized characteristics of participating hospitals are provided in Supplementary Table S1. Both government and private healthcare facilities were included to capture variation in patient experiences across different healthcare settings.

### 2.3 Participants

The target population consisted of adult patients aged 18 years and older who completed their emergency department visit either through discharge or hospital admission. Participants were recruited using a convenience sampling approach immediately after their emergency department encounter and before they left the hospital. Eligible patients were identified by trained research assistants who received standardized instructions regarding participant recruitment and questionnaire administration. Recruitment depended on participant availability, willingness to participate, and the availability of research assistants during the data-collection period; therefore, recruitment was not strictly consecutive. Participation was voluntary, written informed consent was obtained from all respondents, and participants were informed that refusal to participate would not affect the care they received. Detailed recruitment logs, including counts of eligible patients who were not approached or who declined participation, were not systematically maintained.

#### 2.3.1 Inclusion criteria

- Adult patients aged 18 years and above.
- Patients discharged or transferred after ED evaluation.
- Patients capable of providing informed consent.
- Saudi and non-Saudi patients.

#### 2.3.2 Exclusion criteria

- Critically ill or unconscious patients.
- Patients with cognitive impairments.
- Pediatric patients younger than 18 years.

The questionnaire was administered electronically using a structured survey instrument. Patients completed the survey independently whenever possible; however, assistance was provided to participants who experienced difficulty reading or understanding the questions. Surveys were completed only by patients themselves, and accompanying relatives were not permitted to respond on their behalf. Participation was limited to individuals who could understand Arabic, and non-Arabic-speaking patients were therefore excluded from the study.

### 2.4 Population and calculation of sample size

The required sample size was estimated *a priori* using GPower version 3.1 for hierarchical multiple linear regression. The calculation used the “Linear multiple regression: Fixed model, *R*^2^ increase” procedure to evaluate the incremental contribution of the service-quality block after adjustment for demographic and healthcare-related characteristics. Assuming a medium effect size (*f* ^2^ = 0.15), a significance level of 0.05, statistical power of 80%, seven tested service-quality variables, and 14 total predictors in the full model, the minimum required sample size was estimated to be 104 participants. To account for potential non-response and incomplete questionnaires, 400 eligible patients were approached. The GPower analysis yielded seven numerator degrees of freedom, 89 denominator degrees of freedom, a critical *F* value of approximately 2.11, a non-centrality parameter of 15.60, and an actual power of approximately 0.803. The final analytical sample consisted of 313 participants, substantially exceeding the minimum required sample size.

### 2.5 Variables

Overall satisfaction was operationalized as a composite score derived from three five-point Likert-scale items assessing participants' willingness to recommend the hospital, satisfaction with the quality of services received, and perceptions of the functioning of the emergency department. Composite scores were calculated by averaging the responses to the corresponding items, with higher scores indicating greater overall satisfaction. Thus, the dependent variable used in the correlation and regression analyses was a multi-item composite score rather than a single ordinal item. The independent variables consisted of patient-reported perceptions of key dimensions of emergency department service quality, including waiting time, staff availability and responsiveness, patient involvement and communication, clarity of explanation, cleanliness, staff respect, and comfort. In addition, demographic and healthcare-related characteristics were collected and analyzed as potential factors associated with patient satisfaction. These variables included age, gender, marital status, nationality, hospital type, frequency of ED visits during the previous 6 months, and whether the participant received follow-up communication after discharge. For descriptive and inferential analyses, self-reported waiting duration was categorized into three groups (< 2, 2–3, and >3 h) to facilitate an exploratory comparison of overall satisfaction across shorter, intermediate, and longer reported waiting periods. These categories were used as descriptive-analytical groupings and were not intended to represent established triage standards, emergency department performance targets, or clinically validated waiting-time thresholds. For the correlation and regression analyses, overall patient satisfaction was treated as the dependent variable, whereas the service-quality domains and demographic characteristics were treated as independent variables.

### 2.6 Data sources/measurement

Data were collected using a structured self-administered questionnaire distributed to eligible participants following completion of their ED visit. The questionnaire was designed to assess patients' perceptions of the quality of emergency department services and their overall satisfaction with the care received. The survey collected information on demographic characteristics, healthcare utilization patterns, and patient-reported evaluations of key service-quality domains, including waiting time, staff availability and responsiveness, patient involvement and communication, clarity of explanations, cleanliness, respect demonstrated by healthcare staff, comfort of the care environment, and overall satisfaction. All study variables were obtained directly from participants through self-report measures. Responses were recorded using a five-point Likert scale ranging from 1 (extremely dissatisfied) to 5 (extremely satisfied). Standardized data-collection procedures were implemented across all participating sites to ensure consistency and comparability of responses.

### 2.7 Measuring tool/instrument

The study questionnaire consisted of two sections. The first section collected participants' demographic and healthcare-related characteristics, including age, gender, nationality, marital status, hospital type, frequency of emergency department visits, and follow-up contact after discharge.

The second section assessed patients' satisfaction with emergency department services across several domains, including waiting time, staff availability and responsiveness, involvement in decision-making and communication, clarity of explanations, cleanliness, staff respect, and physical comfort. Participants rated each item using a five-point Likert scale ranging from 1 (extremely dissatisfied) to 5 (extremely satisfied).

Overall satisfaction was assessed using three items measuring participants' willingness to recommend the hospital, satisfaction with the quality of services received, and perceptions of the overall functioning of the emergency department. Composite scores for each domain and for overall satisfaction were calculated by averaging the responses to the corresponding items, with higher scores indicating greater satisfaction.

### 2.8 Questionnaire development and validation

The questionnaire was constructed by adapting previously validated patient-experience instruments, including the Press Ganey Emergency Department Patient Experience Survey, SERVQUAL, the Hospital Consumer Assessment of Healthcare Providers and Systems (HCAHPS), and the Saudi Ministry of Health Patient Experience Measurement Program (11, 21–23). Relevant items were selected and modified to reflect the objectives of the present study and the context of Saudi emergency departments.

Content validity was assessed through expert review by specialists in healthcare management and public health. The questionnaire was pilot tested with a small group of participants to assess its clarity, comprehensibility, and appropriateness. Minor revisions were made based on participant feedback before the final questionnaire was administered.

Internal consistency reliability was assessed separately for each questionnaire construct using Cronbach's alpha. Reliability coefficients ranged from 0.76 to 0.85, indicating acceptable to good internal consistency across the multi-item domains. Cronbach's alpha values were 0.84 for staff availability and responsiveness, 0.78 for comfort, 0.81 for clarity of explanation, 0.80 for respect, 0.76 for patient involvement and communication, 0.79 for waiting time, and 0.85 for overall satisfaction. Because cleanliness was assessed using a single item, Cronbach's alpha was not applicable. Complete reliability estimates are presented in Supplementary Table S2.

In addition to assessing reliability, the dimensional structure of the questionnaire was examined using principal component analysis (PCA) with Varimax orthogonal rotation to evaluate whether the questionnaire items clustered according to the pre-defined service-quality domains. Sampling adequacy was assessed using the Kaiser–Meyer–Olkin (KMO) measure and Bartlett's test of sphericity. Factors were retained according to the Kaiser criterion (eigenvalues greater than 1.0), and factor loadings of 0.50 or higher were considered acceptable indicators of item–factor relationships.

### 2.9 Bias

Several measures were implemented to minimize potential sources of bias. Standardized data-collection procedures were applied across all participating sites, and trained research assistants administered the questionnaire using uniform instructions. Participation was voluntary and anonymous to reduce social desirability bias and encourage honest responses. Because satisfaction was assessed using self-reported measures, recall and response biases may have occurred. To minimize these effects, participants were surveyed immediately after completing their ED visit. In addition, the questionnaire was adapted from previously validated patient-satisfaction instruments, which helped reduce measurement bias. Nevertheless, because both the predictor variables and the outcome measure were collected from the same respondents at a single time point, the possibility of common-method variance and response bias cannot be completely excluded.

### 2.10 Statistical methods

Data were analyzed using IBM SPSS Statistics for Windows, Version 26.0 (IBM Corp., Armonk, NY, USA). Descriptive statistics, including frequencies, percentages, means, and standard deviations, were used to summarize participant characteristics and satisfaction scores.

Age was entered into the regression model as a continuous variable measured in years. Although age was presented descriptively in categories to facilitate interpretation, the regression analysis used participants' reported age values. Gender was coded as 0 = male and 1 = female. Hospital type was coded as 0 = government and 1 = private. Nationality was coded as 0 = Saudi and 1 = non-Saudi. Marital status was coded as 0 = single and 1 = married. Follow-up contact was coded as 0 = no and 1 = yes. Visit frequency was entered as an ordinal variable reflecting increasing healthcare utilization.

Pearson correlation analysis was performed to assess associations among the service-quality domains and between each service-quality domain and overall patient satisfaction. One-way analysis of variance (ANOVA) was used to compare overall satisfaction across patient-reported waiting-time categories. *Post hoc* comparisons were conducted using Tukey's Honestly Significant Difference (HSD) test when significant differences were identified. Hierarchical multiple linear regression was performed in two blocks. The first block included demographic and healthcare-utilization variables (age, gender, hospital type, nationality, marital status, visit frequency, and follow-up contact), while the second block additionally included satisfaction with waiting time, staff availability and responsiveness, patient involvement and communication, clarity of explanation, cleanliness, staff respect, and comfort. For each predictor, unstandardized coefficients, standard errors, standardized coefficients, 95% confidence intervals, tolerance values, variance inflation factors, and partial and semi-partial correlations were reported.

Pearson correlation and multiple linear regression were used because the dependent variable was a composite score derived from multiple Likert-scale items rather than a single ordinal item. Multi-item composite scores are commonly treated as approximately continuous when their distributions are adequate and the sample size is sufficient (24, 25).

Prior to conducting inferential analyses, statistical assumptions were evaluated. Linearity, normality, and homoscedasticity were assessed through visual inspection of standardized residual plots, histograms, and normal probability (Q–Q) plots. Homogeneity of variance was examined using Levene's test. Multicollinearity was evaluated using variance inflation factor (VIF) and tolerance statistics. Influential observations were examined using Cook's distance and leverage statistics. Cases with Cook's distance values substantially greater than 1.00 or leverage values exceeding recommended thresholds were considered potentially influential (26). Statistical significance was established at *p* < 0.05.

Questionnaires with more than 50% missing responses were excluded during data screening because they contained insufficient information for reliable interpretation. A total of 400 eligible patients were approached. Sixty declined participation or did not complete the survey. Among the returned questionnaires, 27 were excluded because more than 50% of the questionnaire items were missing. The final analytical sample consisted of 313 participants. For the remaining cases, missing responses were infrequent. Descriptive analyses used all available observations, whereas inferential analyses, including correlation, ANOVA, and regression, were conducted using listwise deletion. A summary of missing values across all study variables is provided in Supplementary Table S3.

### 2.11 Ethical approval

Ethical approval was obtained from the Research Ethics Committee at Saudi Electronic University (Reference: CHS 202510 018). The study was conducted in accordance with the Declaration of Helsinki and applicable local ethical guidelines. Written informed consent was obtained from all participants prior to data collection. Confidentiality, anonymity, and privacy of participant information were maintained throughout the study.

## 3 Results

### 3.1 Participants

**Figure 1:**
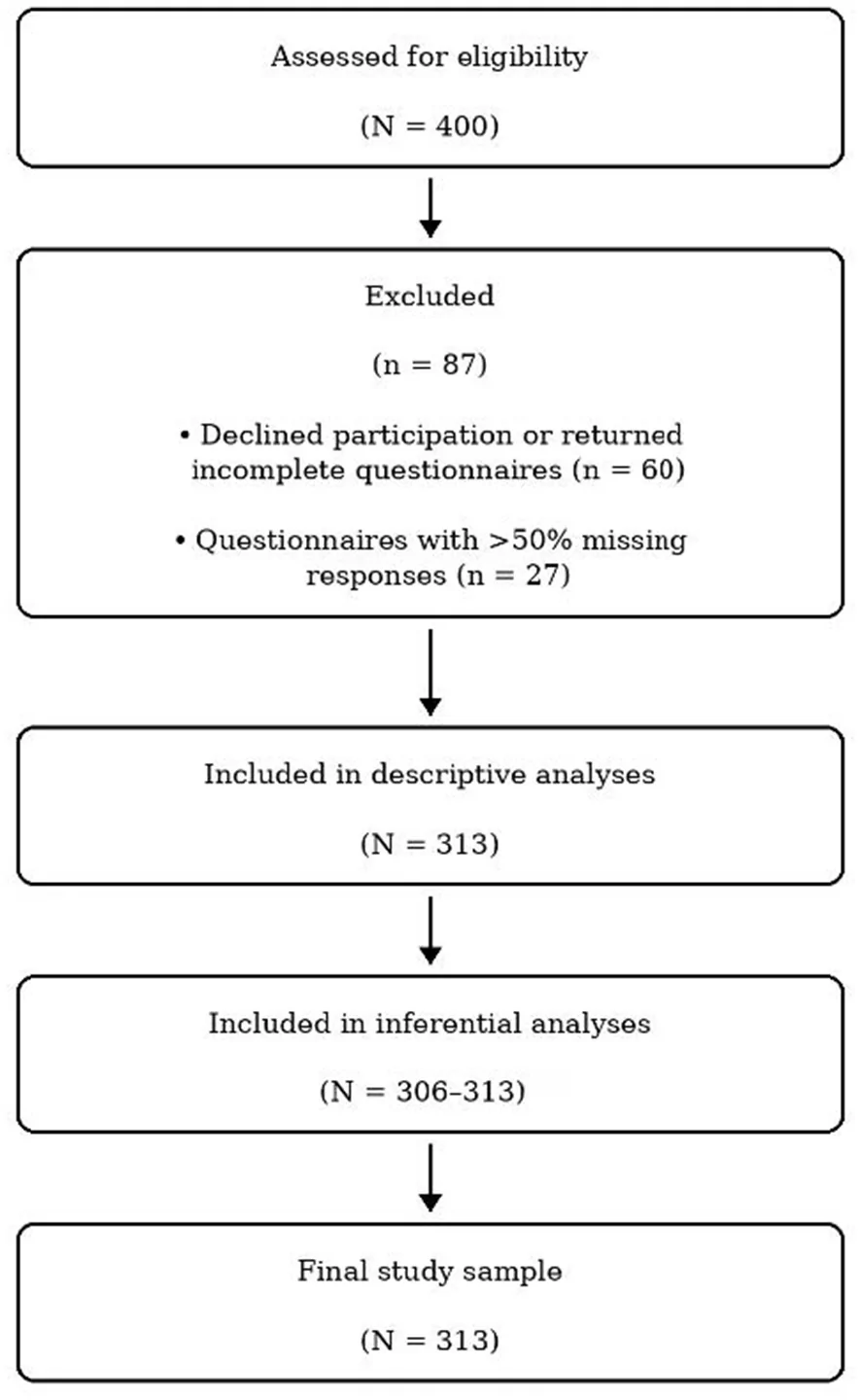
Flow diagram of participant recruitment, exclusion, and inclusion in the analyses.

Data collection was conducted between April 5 and April 23, 2026, in the emergency departments of participating government and private hospitals in Saudi Arabia. A total of 400 eligible patients were approached to participate in the study. Of these, 313 participants completed and returned valid questionnaires, yielding a response rate of 78.3%. Sixty patients declined to participate or did not complete the survey, while 27 questionnaires were excluded because they contained more than 50% missing responses. The final analytical sample therefore consisted of 313 participants (Figure 1).

### 3.2 Descriptive data: demographic characteristics

Female participants represented 76.4% of the sample, while males accounted for 23.6%. The largest age group was 26–35 years (29.7%), followed by 36–45 years (22.4%). Most respondents were Saudi nationals (96.5%) and married (55.6%). Participants recruited from government hospitals accounted for 67.7% of the sample, while 32.3% were recruited from private hospitals. Nearly half of the participants reported one ED visit during the previous 6 months (48.2%), and 36.1% reported receiving follow-up communication after discharge. Additional participant characteristics are presented in Table 1.

**Table 1:** Demographic and healthcare-related characteristics of study participants (*N* = 313).

Characteristic	*n* (%)
Age group	
18–25 years	55 (17.6)
26–35 years	93 (29.7)
36–45 years	70 (22.4)
46–55 years	48 (15.3)
≥56 years	47 (15.0)
Gender	
Female	239 (76.4)
Male	74 (23.6)
Marital status	
Single	139 (44.4)
Married	174 (55.6)
Nationality	
Saudi	302 (96.5)
Non-Saudi	11 (3.5)
Hospital type	
Government	212 (67.7)
Private	101 (32.3)
Emergency department visits (past 6 months)	
One visit	151 (48.2)
Two visits	76 (24.3)
Three visits	48 (15.3)
Four visits	23 (7.3)
Five to nine visits	9 (2.9)
≥10 visits	6 (1.9)
Follow-up contact after discharge	
Yes	113 (36.1)
No	200 (63.9)

Principal component analysis with Varimax rotation supported the construct validity of the questionnaire. The Kaiser–Meyer–Olkin (KMO) measure indicated adequate sampling adequacy (KMO = 0.88), and Bartlett's test of sphericity was statistically significant (χ^2^ = 2,450.6, df = 190, *p* < 0.001), confirming the suitability of the data for principal component analysis. Eight components with eigenvalues greater than 1.0 were retained, explaining 72.4% of the total variance. All questionnaire items demonstrated factor loadings exceeding 0.50 on their respective components, with minimal cross-loadings. Detailed factor loadings, eigenvalues, and the proportion of variance explained by each component are presented in Supplementary Table S4.

Patient satisfaction scores across the evaluated service-quality domains are presented in Table 2. Overall satisfaction with emergency department services was moderate (*M* = 2.79, SD = 1.21). Among the evaluated domains, satisfaction with waiting time received the lowest rating (*M* = 2.46, SD = 1.34), indicating that delays in care remained an important source of dissatisfaction. In contrast, respect demonstrated by healthcare staff received the highest rating (*M* = 3.03, SD = 1.26), suggesting generally favorable perceptions of staff professionalism and interpersonal interactions. The remaining service-quality domains, including staff availability and responsiveness, patient involvement and communication, clarity of explanations, cleanliness, and comfort, exhibited intermediate satisfaction levels, highlighting variation in patients' experiences across different aspects of emergency department care.

**Table 2:** Descriptive statistics for patient service quality among emergency department patients (*N* = 313).

Emergency department service quality	*n*	Mean	SD	Median	IQR
Wait time	313	2.46	1.34	2.00	1–4
Staff availability	313	2.82	1.19	3.00	2–4
Patient involvement and communication	313	2.87	1.20	3.00	2–4
Clarity of explanation	313	2.93	1.27	3.00	2–4
Cleanliness	313	2.97	1.25	3.00	2–4
Staff respect	313	3.03	1.26	3.00	2–4
Comfort	313	2.74	1.24	3.00	2–4
Overall satisfaction	313	2.79	1.21	3.00	2–4

Higher scores indicate greater satisfaction. All items were measured using a 5-point Likert scale ranging from 1 = extremely dissatisfied to 5 = extremely satisfied. IQR, interquartile range; SD, standard deviation.

Reliability analyses demonstrated acceptable to good internal consistency across the questionnaire domains. Cronbach's alpha coefficients for the multi-item constructs ranged from 0.76 to 0.85, indicating satisfactory reliability. The cleanliness domain was assessed using a single item and was therefore excluded from internal consistency analysis. Detailed reliability estimates are presented in Supplementary Table S2.

### 3.3 Outcome data

The primary outcome variable was overall patient satisfaction with emergency department services. Overall satisfaction was operationalized as a composite score derived from three items assessing participants' willingness to recommend the hospital, satisfaction with the quality of services received, and perceptions of the functioning of the emergency department. Composite scores were calculated by averaging the responses to the corresponding items, with higher scores indicating greater overall satisfaction. Descriptive statistics for the outcome variable and the evaluated service-quality domains are presented in Table 2.

### 3.4 Assumption testing

No major violations of the assumptions underlying Pearson correlation, one-way analysis of variance (ANOVA), and multiple linear regression were identified. Visual inspection of histograms, Q–Q plots, and standardized residual plots indicated acceptable normality and homoscedasticity. Levene's test supported the assumption of homogeneity of variance (*p* = 0.24). Multicollinearity was not considered problematic, with variance inflation factor (VIF) values ranging from 1.08 to 2.44 and tolerance values ranging from 0.41 to 0.93. Examination of Cook's distance and leverage statistics did not identify influential observations requiring exclusion.

### 3.5 Main results: correlation analysis

Pearson correlation analysis demonstrated statistically significant positive associations between all evaluated service-quality domains and overall patient satisfaction. Strong correlations were observed for staff availability and responsiveness (*r* = 0.76), staff respect (*r* = 0.75), clarity of explanations (*r* = 0.73), cleanliness (*r* = 0.73), comfort (*r* = 0.72), patient involvement and communication (*r* = 0.70), and satisfaction with waiting time (*r* = 0.67) (all *p* < 0.001). These findings indicate that participants who reported more favorable experiences across the evaluated dimensions of emergency department care also reported higher overall satisfaction with emergency department services. The complete correlation matrix is presented in Supplementary Table S5 and Table 3.

**Table 3:** Pearson correlations between service quality domains and overall patient satisfaction (*N* = 313).

Service quality domain	Pearson's *r*	*p*-value
Satisfaction with waiting time	0.67	< 0.001
Staff availability	0.76	< 0.001
Patient involvement and communication	0.70	< 0.001
Clarity of explanation	0.73	< 0.001
Cleanliness	0.73	< 0.001
Staff respect	0.75	< 0.001
Comfort	0.72	< 0.001

Pearson correlation coefficients represent associations between each service quality domain and overall patient satisfaction. All correlations were statistically significant at *p* < 0.001. Higher correlation coefficients indicate stronger positive associations with overall patient satisfaction.

#### 3.5.1 Comparison of satisfaction across waiting-time categories

A one-way analysis of variance (ANOVA) was conducted to examine differences in overall patient satisfaction across patient-reported waiting-time categories (Table 4). The analysis revealed a statistically significant difference in overall satisfaction among the groups, *F*_(2, 310)_ = 47.83, *p* < 0.001, η^2^ = 0.236. This finding indicates that self-reported waiting-time categories accounted for 23.6% of the variance in overall patient satisfaction within the study sample.

**Table 4:** Overall patient satisfaction according to patient-reported waiting-time category (*N* = 313).

Wait time category	*n*	Mean	SD	95% CI
Short (< 2 h)	89	3.42	0.98	3.21–3.63
Moderate (2–3 h)	124	2.78	1.12	2.58–2.98
Long (>3 h)	100	2.12	1.08	1.91–2.33

One-way ANOVA demonstrated significant differences in overall patient satisfaction across waiting-time categories, *F*_(2, 310)_ = 47.83, *p* < 0.001, η^2^ = 0.236. Tukey's honestly significant difference (HSD) post hoc test indicated statistically significant differences between all pairwise comparisons (all *p* < 0.001). CI, confidence interval; SD, standard deviation.

Levene's test indicated that the assumption of homogeneity of variance was satisfied; therefore, a standard one-way ANOVA and Tukey *post hoc* comparisons were retained (Table 5). *Post hoc* comparisons using Tukey's Honestly Significant Difference (HSD) test revealed statistically significant differences in overall patient satisfaction across all waiting-time categories (Table 6). Patients reporting short waiting times (< 2 h) demonstrated significantly higher satisfaction than those reporting moderate waiting times (mean difference = 0.64, 95% CI: 0.31–0.97, *p* < 0.001) and long waiting times (mean difference = 1.30, 95% CI: 0.95–1.65, *p* < 0.001). Furthermore, patients reporting moderate waiting times reported significantly higher satisfaction than those reporting long waiting times (mean difference = 0.66, 95% CI: 0.35–0.97, *p* < 0.001). Collectively, these findings suggest a progressive decline in patient satisfaction as self-reported waiting duration increases.

**Table 5:** Omnibus ANOVA and homogeneity-of-variance test.

Statistic	Value
*F*-statistic	47.83
Degrees of freedom	(2, 310)
*p*-value	< 0.001
Effect size (η^2^)	0.236
Levene's test statistic	1.42
Levene's test *p*-value	0.24

**Table 6:** *Post hoc* comparisons (Tukey HSD).

Comparison	Mean difference	95% CI	*p*-value
Short vs. moderate	0.64	0.31–0.97	< 0.001
Short vs. long	1.30	0.95–1.65	< 0.001
Moderate vs. long	0.66	0.35–0.97	< 0.001

Exploratory subgroup analyses indicated differences in overall patient satisfaction across selected demographic and hospital-related characteristics. In the unadjusted analyses, female participants, older adults, and patients attending private hospitals reported higher satisfaction scores than their respective comparison groups. However, these findings should be interpreted cautiously because the analyses were exploratory in nature and did not account for multiple comparisons. Complete statistical results are presented in Table 7.

**Table 7:** Exploratory subgroup analyses of overall patient satisfaction according to participant and hospital characteristics.

Characteristic	Category	*n*	Mean (SD)	Test statistic	*p*-value	Effect size	95% CI for mean difference
Gender	Male	74	2.54 (1.18)	*t*(311) = 2.20	0.029	Cohen's *d* = 0.27	0.03, 0.48
	Female	239	2.88 (1.21)				
Age group	18–25 years	55	2.51 (1.15)	*F*_(4, 304)_ = 3.85	0.005	η^2^ = 0.05	—
	26–35 years	93	2.68 (1.18)				
	36–45 years	70	2.82 (1.16)				
	46–55 years	48	3.01 (1.20)				
	≥56 years	43	3.12 (1.22)				
Hospital type	Government	211	2.70 (1.19)	*t*(310) = 2.38	0.018	Cohen's *d* = 0.29	0.05, 0.52
	Private	101	3.05 (1.17)				
Nationality	Saudi	302	2.80 (1.20)	*t*(311) = 0.76	0.448	Cohen's *d* = 0.08	−0.19, 0.42
	Non-Saudi	11	2.68 (1.14)				
Marital status	Single	139	2.74 (1.19)	*t*(308) = 1.21	0.228	Cohen's *d* = 0.14	−0.10, 0.39
	Married	171	2.90 (1.21)				
Follow-up contact	No	195	2.77 (1.20)	*t*(303) = 0.92	0.357	Cohen's *d* = 0.11	−0.11, 0.32
	Yes	110	2.90 (1.18)				

Sample sizes vary across subgroup analyses because inferential analyses were conducted using listwise deletion, and participants with missing values for overall satisfaction or the relevant grouping variable were excluded from the corresponding analysis.

#### 3.5.2 Multiple regression analysis

Regression assumptions were examined before interpretation of the final model and were considered acceptable (see Section 3.4). Table 8 presents the results of the hierarchical regression analysis examining factors associated with overall patient satisfaction.

**Table 8:** Hierarchical multiple regression model summary for overall patient satisfaction.

Model	*R* ^2^	Adjusted *R*^2^	ΔR^2^	*F*_(df1, df2)_	*p*-value	ΔF_(df1, df2)_	*p*-value	Standard error of the estimate
Model 1	0.08	0.06	0.08	3.79 (7, 305)	< 0.001	3.79 (7, 305)	< 0.001	1.14
Model 2	0.58	0.56	0.50	29.40 (14, 298)	< 0.001	50.68 (7, 298)	< 0.001	0.84

In Model 1, demographic and healthcare-utilization variables, including age, gender, hospital type, nationality, marital status, visit frequency, and follow-up contact, explained 8% of the variance in overall satisfaction (*R*^2^ = 0.08, adjusted *R*^2^ = 0.06, *F*_(7, 305)_ = 3.79, *p* < 0.001). After the addition of the service-quality variables in Model 2, the explained variance increased to 58% (*R*^2^ = 0.58, adjusted *R*^2^ = 0.56), representing a statistically significant improvement in model fit (ΔR^2^ = 0.50, Δ*F*_(7, 298)_ = 50.68, *p* < 0.001). The final model was statistically significant overall, *F*_(14, 298)_ = 29.40, *p* < 0.001.

In the fully adjusted model, higher overall patient satisfaction was significantly associated with greater satisfaction with staff availability and responsiveness, comfort, and cleanliness, as well as with receiving care in a private hospital. In contrast, age, gender, nationality, marital status, visit frequency, follow-up contact, satisfaction with waiting time, patient involvement and communication, clarity of explanations, and staff respect were not significantly associated with overall satisfaction after adjustment for the remaining variables. Complete regression coefficients, collinearity statistics, confidence intervals, and partial and semi-partial correlations are presented in Table 9.

**Table 9:** Complete hierarchical regression coefficients for factors associated with overall patient satisfaction (final model).

Variable	B	SE	Standardized β	t	*p*-value	95% CI for B	Partial *r*	Semi-partial *r*	Tolerance	VIF
Age	0.010	0.010	0.05	1.00	0.318	−0.01, 0.03	0.06	0.04	0.82	1.22
Female gender	0.180	0.100	0.08	1.80	0.073	−0.02, 0.38	0.10	0.07	0.85	1.18
Private hospital	0.220	0.110	0.09	2.00	0.046	0.01, 0.43	0.11	0.08	0.80	1.25
Non-Saudi nationality	−0.120	0.190	−0.03	−0.63	0.530	−0.49, 0.25	−0.04	−0.02	0.93	1.08
Married	0.080	0.090	0.04	0.89	0.375	−0.10, 0.26	0.05	0.03	0.88	1.14
Visit frequency	−0.050	0.040	−0.05	−1.25	0.212	−0.13, 0.03	−0.07	−0.04	0.77	1.30
Follow-up contact	0.070	0.090	0.03	0.78	0.438	−0.11, 0.25	0.04	0.03	0.86	1.16
Satisfaction with waiting time	0.090	0.070	0.08	1.29	0.198	−0.05, 0.23	0.07	0.05	0.46	2.17
Staff availability and responsiveness	0.280	0.050	0.28	5.60	< 0.001	0.18, 0.38	0.31	0.22	0.41	2.44
Patient involvement and communication	0.070	0.060	0.06	1.17	0.244	−0.05, 0.19	0.07	0.05	0.49	2.04
Clarity of explanation	0.060	0.060	0.05	1.00	0.318	−0.06, 0.18	0.06	0.04	0.44	2.27
Cleanliness	0.180	0.050	0.19	3.60	< 0.001	0.08, 0.28	0.21	0.14	0.58	1.72
Staff respect	0.050	0.060	0.04	0.83	0.407	−0.07, 0.17	0.05	0.03	0.47	2.13
Comfort	0.240	0.050	0.25	4.80	< 0.001	0.14, 0.34	0.27	0.19	0.55	1.82

Age was entered as a continuous variable. Gender was coded as 0 = male and 1 = female. Hospital type was coded as 0 = government and 1 = private. Nationality was coded as 0 = Saudi and 1 = non-Saudi. Marital status was coded as 0 = single and 1 = married. Follow-up contact was coded as 0 = no and 1 = yes. Visit frequency was coded to reflect increasing numbers of emergency department visits.

### 3.6 Other analyses

Additional subgroup analyses were conducted to examine differences in overall patient satisfaction across demographic and healthcare-related characteristics. Female participants reported significantly higher satisfaction scores than male participants (*p* = 0.029). Satisfaction also differed significantly across age groups, with older adults generally reporting higher levels of satisfaction than younger participants. Furthermore, patients treated in private hospitals reported significantly higher satisfaction than those treated in government hospitals (*p* = 0.018).

No statistically significant differences in overall satisfaction were observed according to nationality or receipt of follow-up communication after discharge (all *p* > 0.05). Although selected demographic and healthcare-setting characteristics were associated with patient satisfaction in these exploratory analyses, the magnitude of these associations appeared to be smaller than those observed for the service-quality variables in the multivariable regression model. These findings should be interpreted cautiously because the analyses were exploratory in nature and did not account for multiple comparisons.

## 4 Discussion

### 4.1 Overview of main findings

This study assessed patient satisfaction with emergency department services and identified factors associated with overall satisfaction among patients attending selected government and private hospitals in Saudi Arabia. Several important findings emerged. First, satisfaction with waiting time received the lowest ratings and was significantly associated with overall satisfaction. Second, staff availability and responsiveness, comfort, and cleanliness emerged as significant independent predictors of overall satisfaction, with staff availability and responsiveness demonstrating the strongest association. Third, satisfaction varied according to hospital type and selected demographic characteristics, with higher levels of satisfaction reported among patients treated in private hospitals and among female participants.

### 4.2 Waiting time and patient satisfaction

The findings demonstrated a clear relationship between self-reported waiting duration and patient satisfaction. Satisfaction with waiting time received the lowest ratings among all evaluated service-quality domains, and overall satisfaction decreased significantly as self-reported waiting duration increased. These findings are consistent with previous studies conducted in Saudi Arabia and internationally, which have identified prolonged waiting times as one of the most important determinants of patient dissatisfaction in emergency department settings (10, 27, 28). Similar concerns have been reported globally, with emergency department crowding and service delays recognized as persistent challenges that adversely affect patient experience and the quality of care (29).

The present findings also align with the psychology of waiting, which suggests that patients' perceptions of delays are shaped not only by the actual duration of waiting but also by uncertainty, anxiety, and the extent to which healthcare providers communicate during the waiting period (30, 31). Consequently, reducing wait times and improving communication about expected delays may be important strategies for enhancing patient satisfaction in emergency departments.

From a theoretical perspective, these findings can be explained by the psychology of waiting. Patients experiencing pain, anxiety, uncertainty, or emotional distress often perceive waiting times as longer than their actual duration, particularly when communication regarding delays is limited (30). Previous research has demonstrated that self-reported waiting duration and the quality of information provided during the waiting process are important determinants of patient satisfaction and may exert a greater influence on patient experience than objective waiting duration (31). Collectively, these findings suggest that, although reducing delays remains important, managing patients' experiences during the waiting period is equally critical for improving satisfaction with emergency department services.

From a theoretical perspective, these findings can be explained by the psychology of waiting. Patients experiencing pain, anxiety, uncertainty, or emotional distress often perceive waiting times as longer than their actual duration, particularly when communication regarding delays is limited (30). Previous research has demonstrated that self-reported waiting duration and the quality of information provided during the waiting process are important determinants of patient satisfaction and may exert a greater influence on patient experience than objective waiting duration (32). Collectively, these findings suggest that, although reducing delays remains important, managing patients' experiences during the waiting period is equally critical for improving satisfaction with emergency department services.

Alternative explanations should also be considered. Staff availability and responsiveness may partly confound or mediate the relationship between waiting experiences and overall satisfaction, because patients who perceive healthcare staff as accessible, attentive, and responsive may evaluate waiting experiences more positively. However, the cross-sectional design and simultaneous measurement of all variables preclude formal tests of mediation and limit the ability to establish temporal ordering or causal relationships. Previous methodological research has demonstrated that cross-sectional analyses may produce biased estimates of mediation effects and cannot adequately capture the dynamic relationships among predictors and outcomes over time (33). Future longitudinal studies incorporating objective operational indicators, such as actual waiting times and staffing levels, together with causal modeling approaches, are needed to clarify the mechanisms linking waiting experiences, service quality, and patient satisfaction.

### 4.3 Comparison with previous studies and theoretical interpretation

The present findings support a growing body of literature demonstrating that patient satisfaction in emergency departments is shaped by multiple operational, interpersonal, and environmental factors (7, 8, 16, 34). At the descriptive level, satisfaction with waiting time received the lowest ratings among the evaluated domains. In the bivariate analyses, all service-quality dimensions were positively correlated with overall satisfaction. After adjustment for demographic characteristics and the remaining service-quality variables, staff availability and responsiveness, comfort, and cleanliness remained significantly associated with overall satisfaction. However, these findings should be interpreted as adjusted statistical associations rather than evidence of causal effects or objective differences in emergency department performance, as the cross-sectional design and simultaneous measurement of predictors and outcomes do not permit causal inference (35).

The distinction between patient perceptions and operational performance is particularly important. Although dissatisfaction with waiting time was prominent, the present study did not collect objective indicators such as actual waiting times, staffing levels, emergency department crowding, or patient volume. Consequently, lower ratings for specific domains should not be interpreted as direct evidence of poorer organizational performance. Rather, the findings reflect patients' subjective evaluations of their emergency department experiences, which represent an important but conceptually distinct dimension of healthcare quality (36, 37).

An important contribution of the present study is the finding that patient-reported service-quality factors explained substantially more variation in overall satisfaction than demographic characteristics alone. While demographic variables explained only a small proportion of satisfaction outcomes, the addition of service-quality variables substantially increased the explained variance. However, part of this increase may reflect shared-method variance and conceptual overlap between some service-quality dimensions and the overall satisfaction measure, because all variables were collected from the same respondents, at the same time, and using the same response format (38, 39).

The findings may also be interpreted through Donabedian's structure–process–outcome framework (15). Within this framework, structural characteristics such as facility cleanliness and comfort, together with process-related factors such as staff availability and responsiveness, influence patient evaluations of healthcare quality and ultimately affect satisfaction outcomes. The fact that staff availability and responsiveness emerged as the strongest predictor suggests that patients place substantial value on accessibility, responsiveness, and timely attention from healthcare professionals, particularly within the high-pressure environment of emergency departments (40). The present findings support the utility of organizing patient-experience measures according to structural and process dimensions of healthcare quality, thereby providing a more integrated understanding of how emergency department characteristics influence satisfaction outcomes (15, 41).

### 4.4 Demographic and hospital type differences

The study identified significant differences in patient satisfaction across hospital types and selected demographic groups. Patients receiving care in private hospitals reported higher satisfaction than those treated in government hospitals. This finding is consistent with previous research suggesting that private healthcare facilities often achieve higher patient satisfaction ratings because of shorter waiting times, greater service flexibility, potentially lower patient-to-provider ratios, and a stronger emphasis on customer-centered service delivery (42, 43). Differences in organizational culture, resource availability, and operational efficiency may also contribute to variations in patient experience across healthcare sectors (41, 44). However, because objective operational indicators, such as actual waiting times, staffing levels, and patient volume, were not available in the present study, these explanations should be interpreted cautiously and warrant further investigation.

Although patients treated in private hospitals reported slightly higher satisfaction, this association should be interpreted cautiously. The effect size was modest, and several potentially important confounding factors, including hospital size, patient acuity, insurance status, staffing levels, admission status, and time of presentation, were not measured. These factors have been shown to influence patient experiences and satisfaction in emergency care settings (7, 35). Consequently, the observed difference should not be interpreted as evidence that private hospitals inherently provide superior emergency department services.

Female participants reported slightly higher satisfaction than male participants. Similar findings have been reported in previous patient satisfaction studies, although the mechanisms underlying these differences remain unclear (7, 25, 26). Potential explanations include differences in healthcare expectations, communication preferences, healthcare utilization patterns, and perceptions of care quality. Nevertheless, the observed gender effect was relatively small and should be interpreted cautiously given the pre-dominance of female respondents in the study sample.

The subgroup analyses and the multivariable regression model yielded partially different findings. For example, although female participants reported higher satisfaction in the unadjusted analyses, gender was not significantly associated with satisfaction after adjustment for service-quality measures and hospital characteristics. This pattern suggests that the observed subgroup differences may have been influenced by confounding factors and differences in patient experiences rather than by demographic characteristics alone, highlighting the importance of multivariable adjustment when interpreting associations in observational studies (35, 45).

### 4.5 Practical and policy implications

The findings of this study suggest that different aspects of emergency department care warrant different levels of priority. First, staff availability and responsiveness, comfort, and cleanliness emerged as the factors most strongly associated with overall satisfaction in the adjusted regression analysis. These domains may therefore represent high-priority targets for quality-improvement initiatives because they remained significantly associated with satisfaction even after accounting for demographic characteristics and other service-quality measures. Consistent with established frameworks for healthcare quality improvement, interventions targeting interpersonal processes and the care environment may contribute to improvements in patient experience and satisfaction outcomes (46).

Second, waiting time received the lowest satisfaction ratings among all evaluated domains, indicating that delays in care remain an important source of dissatisfaction from the patient perspective. However, satisfaction with waiting time was not independently associated with overall satisfaction after adjustment for other service-quality dimensions, suggesting that patients' evaluations of waiting may partly overlap with their perceptions of staff responsiveness, communication, and the care environment. Previous research has shown that patient experiences of waiting are influenced not only by the duration of delays but also by interpersonal interactions, communication, and environmental factors, which may shape overall evaluations of care quality (7, 32).

The present study directly supports interventions aimed at strengthening staff availability and responsiveness and improving the physical care environment, including cleanliness and patient comfort. Because these domains demonstrated significant adjusted associations with overall satisfaction, they may represent important targets for quality-improvement initiatives aimed at enhancing patient experiences (47, 48). However, given the cross-sectional design of the study, these findings should be interpreted as evidence of statistical associations rather than proof that interventions targeting these domains will necessarily improve patient satisfaction.

Additional interventions, such as providing regular updates regarding delays, implementing digital queue-management systems, optimizing workforce planning, and redesigning patient-flow processes, are supported primarily by previous research and healthcare-quality frameworks rather than by the present study alone. Although such strategies may improve patient experience, the current findings cannot establish their effectiveness because objective operational indicators and intervention data were not collected. Consequently, these recommendations should be viewed as evidence-informed strategies that warrant further evaluation in emergency department settings.

From a policy perspective, these findings support ongoing healthcare transformation efforts in Saudi Arabia that emphasize patient-centered and value-based care. Policymakers should interpret the results as evidence of patients' perceptions of service quality rather than as direct measures of emergency department performance (49). Consequently, patient-satisfaction data should be considered alongside objective operational indicators, such as waiting times, staffing levels, and clinical outcomes, when evaluating and improving emergency department services.

The present findings suggest several immediate opportunities for quality improvement in emergency departments. Given the significant associations among staff availability, comfort, cleanliness, and overall satisfaction, healthcare organizations should prioritize interventions that improve the visibility and responsiveness of healthcare professionals, conduct regular environmental cleanliness audits, and enhance patient comfort in waiting and treatment areas. In addition, providing patients with regular updates regarding expected waiting times and service delays may help improve perceptions of care and reduce uncertainty during emergency department visits.

Future quality-improvement initiatives should be accompanied by the routine monitoring of objective operational indicators, including door-to-triage time, door-to-provider time, rates of patients leaving without being seen, and compliance with patient-update protocols. Because these measures were not collected in the present study, the current findings cannot determine their relationship with patient satisfaction.

Additionally, future interventions should be evaluated using pre–post study designs that combine patient-experience measures with objective operational indicators. Such approaches would provide stronger evidence regarding whether improvements in communication, staffing practices, and the emergency department environment lead to measurable changes in patient satisfaction and service performance.

Although these strategies represent plausible approaches to improving emergency department services, the present cross-sectional study did not evaluate their implementation or effectiveness. Consequently, the findings should not be interpreted as evidence that adopting these interventions will necessarily improve patient satisfaction. Prospective studies that combine patient-experience measures with objective operational indicators are needed to determine their impact.

## 5 Limitations

Several limitations should be considered when interpreting the findings of this study.

First, the study relied primarily on patients' self-reported perceptions of their emergency department (ED) experiences. Due to administrative restrictions, objective operational indicators such as actual triage time, physician assessment time, ED length of stay, staffing levels, and patient volume were not available. Consequently, waiting time was assessed on the basis of patient perceptions rather than time-stamped operational records. Perceived waiting time may differ from actual waiting duration and may be influenced by factors such as pain, anxiety, expectations, and communication during the waiting process. Furthermore, because both waiting time and overall satisfaction were self-reported and collected simultaneously, reverse evaluation bias cannot be ruled out. Patients who were dissatisfied with their ED experience may have retrospectively perceived their waiting time as longer, potentially inflating the observed association between waiting and satisfaction.

Second, the cross-sectional study design limits the ability to establish causal relationships between patient satisfaction and the factors examined in this study. Although significant associations were identified between service-quality domains and overall satisfaction, the temporal direction of these relationships cannot be determined. Similarly, because all variables were measured at a single point in time, the study could not determine whether service-quality dimensions, such as staff availability and responsiveness, mediate the relationship between waiting experiences and overall satisfaction. Therefore, the findings should be interpreted as correlational rather than causal.

Third, all service-quality measures and the overall satisfaction outcome were collected from the same respondents, at the same time, and using a common response format immediately following the ED encounter. Consequently, the findings may be susceptible to common-method variance, halo effects, and criterion contamination arising from conceptual overlap between some service-quality dimensions and the overall satisfaction measure. Accordingly, the observed correlations and the proportion of explained variance should not be interpreted as reflecting exclusively substantive relationships. Although exploratory factor analysis provided preliminary evidence regarding the questionnaire structure, the measurement model was not formally validated using confirmatory factor analysis or external validation samples.

Fourth, participants were recruited using convenience sampling from eight hospitals across multiple regions of Saudi Arabia. Recruitment was limited to Arabic-speaking patients, and detailed recruitment logs, site-specific response rates, and information regarding eligible non-participants were not systematically collected. Consequently, selection bias and non-response bias cannot be excluded.

Fifth, the sample's demographic composition may limit the generalizability of the findings. Female participants accounted for 76.4% of the sample, whereas non-Saudi patients represented only 3.5%. The pre-dominance of female respondents may reflect differential participation, recruitment-related factors, or site-specific patterns of emergency department utilization. Similarly, the limited representation of non-Saudi patients restricted the ability to conduct robust nationality comparisons. Because demographic information describing the source populations at participating hospitals was unavailable, it was not possible to determine whether the observed gender distribution accurately reflected emergency department attendance patterns or resulted from selection bias.

Sixth, several important clinical and contextual factors were not included in the analysis. Variables such as triage category, severity of illness, presenting complaint, treatment outcomes, previous healthcare experiences, health literacy, insurance status, socioeconomic characteristics, admission status, and time of presentation may influence patient satisfaction and perceptions of care. In addition, the waiting-time categories used in the analyses (< 2, 2–3, and >3 h) were descriptive groupings rather than clinically validated thresholds or formal ED performance targets. Standardized measures of triage acuity, such as the Emergency Severity Index or equivalent Saudi triage classifications, were not collected. Consequently, the relationship between waiting time and satisfaction could not be interpreted in relation to the clinical appropriateness of waiting times.

Seventh, participants were recruited from eight hospitals; therefore, observations may not have been fully independent because patients were nested within healthcare institutions. Hospital-level characteristics, including ED volume, staffing patterns, crowding, queue-management practices, organizational culture, teaching status, urbanicity, and resource availability, were not systematically documented. Because the study included a limited number of hospitals, multilevel modeling and cluster-robust analyses were not performed. Consequently, standard errors may have been underestimated, and the reported associations should be interpreted cautiously.

Eighth, although hospital type was associated with overall patient satisfaction, several potentially important confounding factors were not measured, including hospital size, staffing levels, ED crowding, patient acuity, and case mix. Therefore, the observed differences between government and private hospitals should not be interpreted as evidence of a causal relationship between hospital sector and patient satisfaction.

Ninth, although the proportion of missing data among retained questionnaires was low, listwise deletion resulted in different effective sample sizes across analyses. Furthermore, because detailed demographic information for excluded respondents was unavailable, it was not possible to formally compare included and excluded participants.

Although measures were taken to protect anonymity and reduce response bias, questionnaires were completed within the hospital setting immediately following the emergency department encounter. Consequently, social-desirability bias and perceived pressure to provide favorable responses cannot be entirely excluded.

In addition, critically ill patients and individuals with cognitive impairment were excluded from participation because they were unable to provide informed consent or complete the questionnaire. Consequently, the study sample may overrepresent medically stable patients and underrepresent individuals with more severe conditions or different patterns of healthcare experiences.

Also, data collection was conducted over a relatively short period of approximately three weeks. As a result, the findings may not fully capture seasonal fluctuations in emergency department utilization, staffing pressures, patient volume, or variations in patient experiences that may occur throughout the year.

Finally, the subgroup analyses were exploratory and conducted without adjustment for multiple comparisons. Consequently, the possibility of type I error cannot be excluded, and these findings should be interpreted cautiously.

Despite these limitations, the study provides valuable evidence regarding patient satisfaction with ED services in Saudi Arabia and identifies several service-quality factors associated with overall satisfaction. The inclusion of participants from multiple hospitals and the use of a structured questionnaire adapted from previously published instruments strengthen the relevance of the findings and provide a foundation for future research and quality-improvement initiatives.

## 6 Conclusions

In this convenience sample drawn from selected government and private hospitals in Saudi Arabia, patient satisfaction with emergency department services varied across multiple dimensions of care. Staff availability and responsiveness, comfort, cleanliness, and hospital sector were independently associated with overall satisfaction in the adjusted model, whereas waiting time emerged as the lowest-rated aspect of the patient experience.

These findings highlight several potentially modifiable domains that may influence patients' perceptions of emergency department care. However, the results should be interpreted as exploratory and context-specific rather than as representative of all emergency departments in Saudi Arabia. Future research should employ representative multicentre designs, incorporate objective operational indicators and validated measurement models, and account for hospital-level clustering to better understand the factors associated with patient satisfaction and to evaluate potential quality-improvement strategies.

## Data Availability

The raw data supporting the conclusions of this article will be made available by the authors, without undue reservation.
