# Supplementary material for: Patient satisfaction with emergency department services in selected Saudi hospitals: a cross-sectional study
Source: Front Med (Lausanne). 2026 Aug 19;13:1900551. doi: 10.3389/fmed.2026.1900551 (PMC13533912; doi:10.3389/fmed.2026.1900551)
Supplement: Supplementary file 1 [file Data_Sheet_1.docx]

**Patient Satisfaction with Emergency Department Services in Selected Saudi Hospitals: A Cross-Sectional**

**Questionnaire**

The questionnaire used in this study was developed by adapting concepts from the Press Ganey Emergency Department Patient Experience Survey, SERVQUAL, the Hospital Consumer Assessment of Healthcare Providers and Systems (HCAHPS), and the Saudi Ministry of Health Patient Experience Measurement Program. The items were substantially modified and used only as conceptual sources to inform the development of the study instrument rather than being reproduced in their original form. Consequently, no formal permission was required from the original instrument developers.

**Section A. Demographic Information**

**Age:**

18–25

26–35

36–45

46–55

56 and above

**Gender**:

Male

Female

**Nationality:**

Saudi

Non-Saudi

**Education level:**

Primary or below

Secondary

Diploma

Bachelor’s degree

Postgraduate degree

**Type of hospital:**

Public

Private

**Number of visits to the ED in the preceding 6 months:**

1 time

2 times

3 times

4 times

5-9 times

≥10 times

**Follow-Up Contact After Discharge**

Yes

No

**Section B. Emergency Department Service Quality**

**Please indicate your level of satisfaction with each aspect of emergency department services using the following scale:**

1 = Extremely dissatisfied
2 = Dissatisfied
3 = Neutral
4 = Satisfied
5 = Extremely satisfied

**1. Staff Availability and Responsiveness (6 items)**

- Nurses care about my treatment.
- Nurses inform me about the remaining part of my treatment.
- Nurses attend to me patiently.
- Nurses relieve my pain well.
- Admission staff guide me appropriately.
- The behavior of the admission staff is suitable.

**2. Comfort (2 items)**

- The environment of the emergency room is calm and quiet.
- The emergency room is well equipped.

**3. Cleanliness (1 item)**

- The environment of the emergency room is hygienic.

**4. Clarity of Explanation (2 items)**

- The physician explained my treatment course.
- The physician’s explanation about the remaining treatment was clear enough.

**5. Respect (2 items)**

- The behavior of the physician was respectful.
- The family of the patient is respected in this hospital.

**6. Patient Involvement and Communication (2 items)**

- The physician spent sufficient time examining me.
- Family members can spend an appropriate amount of time beside the patient.

**7. Waiting Time (2 items)**

- The waiting time before seeing the doctor was appropriate.
- The waiting time before the admission process was appropriate.

**8. Overall Satisfaction (3 items)**

- I would recommend this hospital to my acquaintances.
- I am satisfied with the quality of services in the emergency room.
- The emergency room of this hospital is well functioning.

**Note:** For statistical analysis, conceptually related items were combined into composite variables representing staff availability and responsiveness, comfort, cleanliness, clarity of explanation, respect, patient involvement and communication, waiting time, and overall satisfaction. Composite scores were calculated by averaging the responses to the corresponding items, with higher scores indicating greater satisfaction.
