# Supplementary material for: Patient satisfaction with emergency department services in selected Saudi hospitals: a cross-sectional study
Source: Front Med (Lausanne). 2026 Aug 19;13:1900551. doi: 10.3389/fmed.2026.1900551 (PMC13533912; doi:10.3389/fmed.2026.1900551)
Supplement: Supplementary file 2 [file Data_Sheet_2.docx]

**رضا المرضى عن خدمات أقسام الطوارئ في مستشفيات سعودية مختارة: دراسة مقطعية**

**الاستبيان**

**القسم الأول: المعلومات الديموغرافية**

العمر:

- 18–25 سنة
- 26–35 سنة
- 36–45 سنة
- 46–55 سنة
- 56 سنة فأكثر

الجنس:

- ذكر
- أنثى

الجنسية:

- سعودي
- غير سعودي

المستوى التعليمي:

- ابتدائي أو أقل
- ثانوي
- دبلوم
- بكالوريوس
- دراسات عليا

نوع المستشفى:

- حكومي
- خاص

عدد الزيارات لقسم الطوارئ خلال الأشهر الستة الماضية:

- مرة واحدة
- مرتان
- ثلاث مرات
- أربع مرات
- من 5 إلى 9 مرات
- 10 مرات أو أكثر

التواصل بعد الخروج من المستشفى:

- نعم
- لا

**القسم الثاني: جودة خدمات قسم الطوارئ**

يرجى تحديد مستوى رضاك عن كل جانب من جوانب خدمات قسم الطوارئ باستخدام المقياس التالي:

1 = غير راضٍ إطلاقًا

2 = غير راضٍ

3 = محايد

4 = راضٍ

5 = راضٍ جدًا

1. توفر واستجابة الطاقم الطبي (6 بنود)

- يهتم طاقم التمريض بعلاجي.
- يقوم طاقم التمريض بإبلاغي بالمراحل المتبقية من علاجي.
- يتعامل طاقم التمريض معي بصبر.
- يخفف طاقم التمريض من ألمي بشكل جيد.
- يرشدني موظفو الاستقبال والإدخال بشكل مناسب.
- سلوك موظفي الاستقبال والإدخال مناسب.

2. الراحة (بندان)

- بيئة قسم الطوارئ هادئة ومريحة.
- قسم الطوارئ مجهز تجهيزًا جيدًا.

3. النظافة (بند واحد)

- بيئة قسم الطوارئ نظيفة وصحية.

4. وضوح الشرح (بندان)

- شرح الطبيب لي خطة علاجي.
- كان شرح الطبيب للمراحل المتبقية من العلاج واضحًا بما فيه الكفاية.

5. الاحترام (بندان)

- كان سلوك الطبيب محترمًا.
- تُعامل أسرة المريض باحترام في هذا المستشفى.

6. مشاركة المريض والتواصل (بندان)

- خصص الطبيب وقتًا كافيًا لفحصي.
- يُسمح لأفراد الأسرة بقضاء وقت مناسب بجانب المريض.

7. وقت الانتظار (بندان)

- كان وقت الانتظار قبل مقابلة الطبيب مناسبًا.
- كان وقت الانتظار قبل إتمام إجراءات الدخول مناسبًا.

8. الرضا العام (3 بنود)

- أوصي بهذا المستشفى لأقاربي ومعارفي.
- أنا راضٍ عن جودة الخدمات المقدمة في قسم الطوارئ.
- يعمل قسم الطوارئ في هذا المستشفى بكفاءة جيدة.
