# Supplementary material for: Patient satisfaction with emergency department services in selected Saudi hospitals: a cross-sectional study
Source: Front Med (Lausanne). 2026 Aug 19;13:1900551. doi: 10.3389/fmed.2026.1900551 (PMC13533912; doi:10.3389/fmed.2026.1900551)
Supplement: Supplementary file 3 [file Table_1.docx]

**Supplementary Table S1. Characteristics of Participating Hospitals**

| **Hospital** | **Sector** | **Region** | **Hospital Type** | **Urban Setting** |
| --- | --- | --- | --- | --- |
| Hospital A | Government | Central | General hospital | Yes |
| Hospital B | Government | Central | General hospital | Yes |
| Hospital C | Government | Western | General hospital | Yes |
| Hospital D | Government | Eastern | General hospital | Yes |
| Hospital E | Government | Western | General hospital | Yes |
| Hospital F | Private | Central | Private hospital | Yes |
| Hospital G | Private | Eastern | Private hospital | Yes |
| Hospital H | Private | Western | Private hospital | Yes |

*Note. Hospital identities are anonymized to preserve institutional confidentiality. Information regarding emergency department volume, teaching status, site-specific participant contributions, and recruitment by shift was not systematically collected during the study and is therefore unavailable..*
