# Supplementary material for: Patient satisfaction with emergency department services in selected Saudi hospitals: a cross-sectional study
Source: Front Med (Lausanne). 2026 Aug 19;13:1900551. doi: 10.3389/fmed.2026.1900551 (PMC13533912; doi:10.3389/fmed.2026.1900551)
Supplement: Supplementary file 4 [file Table_2.docx]

**Supplementary Table S2. Internal Consistency Reliability of Questionnaire Constructs**

| **Construct** | **Number of Items** | **Cronbach’s α** |
| --- | --- | --- |
| Staff availability and responsiveness | 6 | 0.84 |
| Comfort | 2 | 0.78 |
| Cleanliness | 1 | Not applicable |
| Clarity of explanation | 2 | 0.81 |
| Respect | 2 | 0.80 |
| Patient involvement and communication | 2 | 0.76 |
| Waiting time | 2 | 0.79 |
| Overall satisfaction | 3 | 0.85 |

**Note.** Cronbach’s alpha coefficients are reported separately for each construct. The cleanliness construct consisted of a single item; therefore, internal consistency reliability could not be assessed using Cronbach’s alpha. Values shown above are illustrative placeholders and should be replaced with the actual coefficients obtained from the study data.
