# Supplementary material for: Patient satisfaction with emergency department services in selected Saudi hospitals: a cross-sectional study
Source: Front Med (Lausanne). 2026 Aug 19;13:1900551. doi: 10.3389/fmed.2026.1900551 (PMC13533912; doi:10.3389/fmed.2026.1900551)
Supplement: Supplementary file 5 [file Table_3.docx]

**Supplementary Table S3. Exploratory Factor Analysis of Questionnaire Items**

| **Construct** | **Number of items** | **Eigenvalue** | **Variance explained (%)** | **Factor loading range** |
| --- | --- | --- | --- | --- |
| Staff availability and responsiveness | 6 | 4.82 | 24.1 | 0.66–0.84 |
| Comfort | 2 | 2.14 | 10.7 | 0.71–0.80 |
| Cleanliness | 1 | — | — | 0.77 |
| Clarity of explanation | 2 | 1.76 | 8.8 | 0.69–0.83 |
| Respect | 2 | 1.53 | 7.6 | 0.73–0.86 |
| Patient involvement and communication | 2 | 1.34 | 6.7 | 0.62–0.79 |
| Waiting time | 2 | 1.18 | 5.9 | 0.68–0.81 |
| Overall satisfaction | 3 | 1.71 | 8.6 | 0.74–0.88 |
