# Supplementary material for: Patient satisfaction with emergency department services in selected Saudi hospitals: a cross-sectional study
Source: Front Med (Lausanne). 2026 Aug 19;13:1900551. doi: 10.3389/fmed.2026.1900551 (PMC13533912; doi:10.3389/fmed.2026.1900551)
Supplement: Supplementary file 7 [file Table_5.docx]

**Supplementary Table S5. Correlation Matrix for Study Variables (N = 313)**

| **Variable** | **1** | **2** | **3** | **4** | **5** | **6** | **7** | **8** | **9** | **10** | **11** | **12** | **13** | **14** | **15** |
| --- | --- | --- | --- | --- | --- | --- | --- | --- | --- | --- | --- | --- | --- | --- | --- |
| **1. Age** | **1.00** |  |  |  |  |  |  |  |  |  |  |  |  |  |  |
| **2. Gender** | **0.04** | **1.00** |  |  |  |  |  |  |  |  |  |  |  |  |  |
| **3. Hospital type** | **0.08** | **0.06** | **1.00** |  |  |  |  |  |  |  |  |  |  |  |  |
| **4. Nationality** | **-0.03** | **0.02** | **0.05** | **1.00** |  |  |  |  |  |  |  |  |  |  |  |
| **5. Marital status** | **0.41** | **0.01** | **0.03** | **-0.02** | **1.00** |  |  |  |  |  |  |  |  |  |  |
| **6. Visit frequency** | **0.09** | **0.07** | **0.11** | **0.01** | **0.08** | **1.00** |  |  |  |  |  |  |  |  |  |
| **7. Follow-up contact** | **0.06** | **0.04** | **0.09** | **-0.01** | **0.05** | **0.14** | **1.00** |  |  |  |  |  |  |  |  |
| **8. Waiting time** | **0.12** | **0.08** | **0.19** | **0.02** | **0.06** | **0.13** | **0.10** | **1.00** |  |  |  |  |  |  |  |
| **9. Staff availability** | **0.09** | **0.11** | **0.16** | **0.03** | **0.08** | **0.12** | **0.14** | **0.62** | **1.00** |  |  |  |  |  |  |
| **10. Patient involvement** | **0.08** | **0.09** | **0.14** | **0.04** | **0.07** | **0.10** | **0.13** | **0.58** | **0.73** | **1.00** |  |  |  |  |  |
| **11. Clarity** | **0.07** | **0.10** | **0.12** | **0.01** | **0.05** | **0.09** | **0.12** | **0.55** | **0.70** | **0.76** | **1.00** |  |  |  |  |
| **12. Cleanliness** | **0.06** | **0.08** | **0.18** | **0.03** | **0.04** | **0.07** | **0.10** | **0.53** | **0.68** | **0.67** | **0.72** | **1.00** |  |  |  |
| **13. Respect** | **0.05** | **0.09** | **0.14** | **0.02** | **0.06** | **0.08** | **0.11** | **0.57** | **0.74** | **0.75** | **0.73** | **0.71** | **1.00** |  |  |
| **14. Comfort** | **0.08** | **0.07** | **0.17** | **0.01** | **0.05** | **0.09** | **0.11** | **0.56** | **0.71** | **0.70** | **0.69** | **0.68** | **0.72** | **1.00** |  |
| **15. Overall patient satisfaction** | **0.10** | **0.11** | **0.20** | **0.02** | **0.08** | **0.09** | **0.10** | **0.67** | **0.76** | **0.70** | **0.73** | **0.73** | **0.75** | **0.72** | **1.00** |

**Note: Values represent Pearson correlation coefficients. Gender was coded as male = 0 and female = 1; hospital type as government = 0 and private = 1; nationality as non-Saudi = 0 and Saudi = 1; marital status as single = 0 and married = 1; and follow-up contact as no = 0 and yes = 1. Correlations involving overall patient satisfaction correspond to the results reported in Table 3. Values shown above are illustrative placeholders and should be replaced with the exact output from SPSS before submission.**

*** p < 0.05.
** p < 0.01.
*** p < 0.001.**
