# Supplementary material for: Patient satisfaction with emergency department services in selected Saudi hospitals: a cross-sectional study
Source: Front Med (Lausanne). 2026 Aug 19;13:1900551. doi: 10.3389/fmed.2026.1900551 (PMC13533912; doi:10.3389/fmed.2026.1900551)
Supplement: Supplementary file 8 [file Table_6.docx]

Supplementary Table S6. Mapping of Questionnaire Items to Study Variables

| Study Variable | Questionnaire Items | Number of Items |
| --- | --- | --- |
| Staff Availability and Responsiveness | Nurses care about my treatment. Nurses inform me about the remaining part of my treatment. Nurses attend to me patiently. Nurses relieve my pain well. Admission staff guide me appropriately. The behavior of the admission staff is suitable. | 6 |
| Comfort | The environment of the emergency room is calm and quiet. The emergency room is well equipped. | 2 |
| Cleanliness | The environment of the emergency room is hygienic. | 1 |
| Clarity of Explanation | The physician explained my treatment course. The physician’s explanation about the remaining treatment was clear enough. | 2 |
| Respect | The behavior of the physician was respectful. The family of the patient is respected in this hospital. | 2 |
| Communication and Engagement | The physician spent sufficient time examining me. Family members can spend an appropriate amount of time beside the patient. | 2 |
| Waiting Time | The waiting time before seeing the doctor was appropriate. The waiting time before the admission process was appropriate. | 2 |
| Overall Satisfaction | I would recommend this hospital to my acquaintances. I am satisfied with the quality of services in the emergency room. The emergency room of this hospital is well functioning. | 3 |

Note. The final questionnaire consisted of 20 items organized into eight conceptual constructs, in addition to demographic questions. Questionnaire items were adapted from the Press Ganey Emergency Department Patient Experience Survey, SERVQUAL, the Hospital Consumer Assessment of Healthcare Providers and Systems (HCAHPS), and the Saudi Ministry of Health Patient Experience Measurement Program. The items were substantially modified and grouped into conceptually related composite variables representing operational, interpersonal, and environmental aspects of emergency department care. Composite scores were calculated by averaging the responses to the corresponding items, with higher scores indicating greater satisfaction. Overall satisfaction was treated as the primary outcome variable.
